# GDF-15 plasma levels are elevated in mobility-limited older adults with frailty and sarcopenia—results from the BIOFRAIL study

**DOI:** 10.1007/s11357-025-01946-6

**Published:** 2025-11-25

**Authors:** Pernille Hansen, Hanne Nygaard, Louis Praeger-Jahnsen, Martin Schultz, Flemming Dela, Per Aagaard, Jesper Ryg, Charlotte Suetta

**Affiliations:** 1 Geriatric Research Unit, Department of Medicine, Copenhagen University Hospital, Herlev and Gentofte, Gentofte, Denmark; 2 Geriatric Research Unit, Department of Geriatric and Palliative Medicine, Copenhagen University Hospital, Bispebjerg and Frederiksberg, Copenhagen, Denmark; 3 Institute of Clinical Medicine, Faculty of Health and Medical Sciences, CopenAge - Copenhagen Center for Clinical Age Research, University of Copenhagen, Copenhagen, Denmark; 4 Department of Emergency Medicine, Copenhagen University Hospital, Bispebjerg and Frederiksberg, Copenhagen, Denmark; 5 Copenhagen Center for Translational Research, Copenhagen University Hospital, Bispebjerg and Frederiksberg, Copenhagen, Denmark; 6 Institute of Clinical Medicine, Faculty of Health, University of Copenhagen, Copenhagen, Denmark; 7 Department of Biomedical Sciences, Faculty of Health and Medical Sciences, University of Copenhagen, Copenhagen, Denmark; 8 Department of Sports Sciences and Clinical Biomechanics, University of Southern Denmark, Odense, Denmark; 9 Geriatric Research Unit, Department of Clinical Research, University of Southern Denmark, Odense, Denmark; 10 Department of Internal Medicine Geriatric Section, Copenhagen University Hospital, Herlev and Gentofte, Herlev, Denmark

**Keywords:** Biomarker, Frailty, GDF-15, Mobility-Limited Patients, Old Age, Sarcopenia

## Abstract

Growth differentiation factor 15 (GDF-15) has been proposed as a potential biomarker for the geriatric syndromes of frailty and sarcopenia. This study investigated plasma GDF-15 as a biomarker associated with sarcopenia and frailty in a cohort of mobility-limited older adults. Patients (≥ 65 years) were recruited from a geriatric outpatient clinic for fall assessment. Frailty was identified using the Clinical Frailty Scale with a cut-off ≥ 5. Sarcopenia was defined according to the European Working Group on Sarcopenia in Older People 2. Plasma GDF-15 was analyzed using electrochemiluminescence assays (Meso Scale Discovery, Rockville, MD, USA). Receiver operating characteristic (ROC) analysis was performed to assess the diagnostic accuracy of GDF-15 for identifying frailty and sarcopenia. The optimum GDF-15 cut-off for frailty was determined using Youden’s Index. A total of 429 patients (age 79.6 ± 6.2 years, 64% female) were included. Of these, 16% had sarcopenia and 25% had frailty. Median GDF-15 levels in patients with/without sarcopenia and with/without frailty were 1916 pg/mL vs. 1569 pg/mL (p = 0.035), and 2252 pg/mL vs. 1438 pg/mL (p < 0.001), respectively. The area under the curve (AUC) for frailty was 0.681 (95% CI: 0.623–0.739) with an optimal cut-off of 2047 pg/mL. For sarcopenia, AUC was 0.577 (95% CI: 0.501–0.653). GDF-15 plasma levels appear to be elevated in mobility-limited older adults with frailty and sarcopenia. The diagnostic accuracy of GDF-15 for frailty appears of moderate magnitude, suggesting a potential clinical role of this biomarker in identifying frailty in mobility-limited older adults. In contrast, the diagnostic accuracy of GDF-15 for sarcopenia was poor. ClinicalTrials.gov Identifier: NCT05795556.

**Supplementary Information:**

The online version contains supplementary material available at 10.1007/s11357-025-01946-6.

## Background

The global population is aging and the proportion of older adults is expected to continue to increase during the forthcoming decades [1]. This demographic shift is paralleled by an increased prevalence of age-related conditions, including frailty and sarcopenia.

Frailty is defined as a clinical syndrome associated with increased vulnerability to adverse health outcomes, including falls, hospitalisation, and mortality [2, 3]. The prevalence of frailty ranges between 2.9–77.0% depending on the population studied and the assessment tools used [4, 5]. Consequently, there is growing interest in using circulating biomarkers as early indicators of frailty, as well as their potential as objective and quantifiable measures of physiological decline [6]. An initial broad characterization of potential biomarkers for frailty, including hormones, inflammatory markers, and nutritional biomarkers such as vitamins and minerals, was presented by Ferrucci and colleagues in 2002 [7]. However, the predictive strength (specificity) of selected biomarkers for frailty has not been extensively investigated [6].

Sarcopenia, which is defined as a progressive loss of muscle strength, muscle mass, and function, also increases the risk of negative outcomes such as falls, fractures, and mortality [8, 9]. Among community-dwelling older adults, the prevalence of sarcopenia ranges from 10–27% depending on the population and the classification used [10, 11]. Due to the complex nature of sarcopenia, the European Working Group on Sarcopenia in Older People (EWGSOP) has suggested the identification of potential biomarkers to diagnose and monitor older people with sarcopenia [8].

Growth Differentiation Factor-15 (GDF-15), a member of the transforming growth factor-beta (TGF-β) superfamily [12], is a stress-responsive cytokine that has gained increasing interest as a promising biomarker of biological aging and age-related diseases [13–15]. GDF-15 is markedly upregulated in plasma during aging and plays a central role in the human stress response, reflecting systemic effects of cellular damage, inflammation, and metabolic dysfunction [14, 16, 17]. Moreover, elevated GDF-15 plasma levels have been associated with a wide range of chronic conditions, including cardiovascular disease [18], cancer [19], diabetes [20, 21], and chronic kidney disease [22]. GDF-15 is also known to be elevated in conditions characterized by muscle atrophy and weakness, and it is hypothesized to play a pivotal role in the decline of functional performance with aging [23, 24]. Several studies have demonstrated an association between elevated GDF-15 plasma levels and reduced muscle performance as well as increased inflammation in patients with lower-limb mobility limitations [25, 26].

Although GDF-15 has been proposed as a potential biomarker for both sarcopenia and frailty [24, 27], the role of GDF-15 may differ substantially between the above conditions. Further, the clinical applicability of plasma GDF-15 assessments in older patients remains underexplored. Given its feasibility in clinical settings, GDF-15 plasma level could potentially serve as a supplementary marker to existing clinical assessments. Furthermore, if GDF-15 plasma levels are less influenced by variations across patient populations, it may hold particular clinical relevance as a stable biomarker for identifying frailty and sarcopenia in diverse settings. Thus, the present study aimed to investigate the association between GDF-15 plasma levels and the presence of frailty and sarcopenia in mobility-limited older adults referred to an outpatient clinic for fall assessment.

## Methods

### Setting and patients

The current study is based on the BIOFRAIL study, a Copenhagen-based cross-sectional cohort study, which included 508 patients aged ≥ 65 years referred to the geriatric outpatient fall clinic at Copenhagen University Hospital, Herlev and Gentofte, Denmark between September 2021 and June 2023. The study is reported according to STROBE (Strengthening the Reporting of Observational Studies in Epidemiology) guidelines [28].

The study was approved by the Health Research Ethics Committee of Copenhagen and Frederiksberg (H-20057620), and the Danish Data Protection Agency (P-2021–619), and conducted in accordance with the Declaration of Helsinki. All included patients gave their written informed consent before inclusion.

Briefly, patients were referred to the outpatient clinic for a fall assessment with an age of 65 years or above to be considered eligible to participate in the study. Exclusion criteria were: severe dementia, inability to understand or adhere to the test protocol, communicative problems, inability to provide informed consent, and inability to walk independently without personal support (walking aids allowed).

### Frailty

Frailty was assessed using the 9-point Clinical Frailty Scale (CFS), with a cut-off CFS ≥ 5 for dichotomous identification of frailty [29]. Additionally, patients were categorized into three groups based on their CFS score: non-frail (CFS 1–3), frail (CFS 4–5), and severely frail (CFS ≥ 6) [30].

### Sarcopenia

Sarcopenia was assessed according to guidelines defined by the European Working Group on Sarcopenia in Older People (EWGSOP2) [8]. Pre-sarcopenia was defined as low muscle strength only; sarcopenia was defined as low muscle strength and reduced muscle mass; and severe sarcopenia was defined as low muscle strength, reduced muscle mass, and low physical performance [8].

Low muscle strength was defined as at least one of the following three criteria: handgrip strength < 27 kg for males and < 16 kg for females, 30-s sit-to-stand (STS) ≤ 8 repetitions, and/or 5-reps STS > 15 s [8, 31]. Low muscle mass was defined as skeletal muscle index (SMI) < 7.0 kg/m^2^ for males and < 5.5 kg/m^2^ for females, while low physical performance was defined as a habitual gait speed (GS) ≤ 0.8 m/s [8].

### Handgrip strength

Maximal isometric handgrip strength was measured using a hand-held dynamometer (Jamar Smart, Sammons Preston Rolyan, Chicago, Illinois, USA) with three trials performed with the dominant hand interspaced by a short pause (30 s) between successive trials [32]. If strength increased in the third attempt, additional trials were performed until no further improvements could be observed. Patients were seated in a rigid chair; the shoulder adducted and neutrally rotated; the elbow had to be at a 90-degree angle; the wrist in a neutral position; and the forearm supported by armrests. When ready, the patient was instructed to squeeze the dynamometer with maximum effort, for approximately 3 s, ensuring no additional body movement occurred. The trial with the highest peak force was selected for further analysis.

### Sit-to-stand performance

The 30-s STS test measures the number of STS repetitions performed by the patients in 30 s, and the 5-reps STS test measures the time taken to complete five full STS cycles from a sitting position. In the starting position, patients were seated in the middle of a chair, back straight, feet approximately a shoulder width apart, and arms across the chest [33, 34]. The chair was positioned against a wall to ensure stability during the tests. At the signal “go,” the patients raised from the chair to a full stand (body straight and upright) and then returned to the starting seated position [33, 34]. Patients were instructed to perform the STS repetitions as rapidly as possible.

A stopwatch was started simultaneously with the “go” cue and stopped either after the 30-s time limit elapsed or after the completion of five repetitions, respectively. The final score reflects the total number of full stands (more than halfway up at the end of the 30-s counted as a full stand) [34]. Additionally, the time taken to complete five full stands was recorded to the nearest 0.01 s [33].

### Muscle mass

Appendicular muscle mass was assessed using direct segmental multi-frequency bioelectrical impedance analyses (BIA) (Inbody770, InbodyS10; Biospace Co., Seoul, Korea) and reported as SMI, calculated as the ratio of appendicular lean mass to height squared (kg/m^2^). Additionally, muscle mass was adjusted using the equation derived by Kristensen et al. [35] for older adults to account for the slight systematic overestimation of ALM by BIA compared to DXA:

$$ALM*h-2 \left(DXA\right)\hspace{0.17em}=\hspace{0.17em}5.03\hspace{0.17em}+\hspace{0.17em}1.17 * ALM*h-2 \left(BIA\right)- 0.039 * body height$$

### Gait speed

Habitual GS was measured over a 6 m distance, with an acceleration and deceleration phase of 1–1.5 m each. Time was recorded using a stopwatch, which was started when the patient’s one foot (toe) crossed the starting line and stopped when one foot crossed the finish line. Patients were instructed to walk at a normal pace, and usual walking aids were allowed. The patients performed two attempts, and GS (m/s) was calculated as the distance divided by the fastest time recorded [36].

### Patient-reported outcomes

Information on any falls within the past year was collected using self-reported schemes and categorized into three groups: no falls, 1–3 falls, or ≥ 4 falls.

Malnutrition risk was assessed using the Short Nutritional Assessment Questionnaire (SNAQ) [37] with a score of < 2 points indicating well-nourished status, a score of 2 reflecting moderate malnutrition, and scores ≥ 3 indicating severe malnutrition [37].

### GDF-15 plasma levels

Blood samples were drawn from an antecubital vein and immediately placed on ice in an upright position in a designated rack. The separation process was performed at 3172 × g at 4 °C for 10 min. Plasma was then extracted and immediately frozen at -80 °C until further analysis.

GDF-15 plasma levels were analysed using R-PLEX electrochemiluminescence assays (Meso Scale Discovery, Rockville, MD, USA) and analysed according to the manufacturer’s protocol, with a few alterations. GDF-15 plasma levels were measured over a three-day period. Plasma samples were diluted 150-fold, initially in Diluent 100 and subsequently in assay diluent. A control sample was included on each plate, yielding a mean concentration of 248.11 pg/mL, with a standard deviation of 40.80 pg/mL and a coefficient of variation (CV) of 16.36% across all plates.

### Statistical analysis

Visually inspection through residual plots and histograms was used to evaluate the normality of distribution. Descriptive statistics were conducted to assess relative percentages, as well as mean or median values based on the data distribution.

Patients’ characteristics are presented as a total group mean, followed by differences between the frail and non-frail subgroups and differences between the sarcopenic and non-sarcopenic subgroups. Independent *t*-tests were used for normally distributed variables, Mann–Whitney U tests for non-normally distributed variables, and chi-squared tests for categorical variables to investigate potential between-group differences.

Differences in GDF-15 plasma levels across frailty and sarcopenia categories were analysed using the Kruskal–Wallis test for independent samples and adjusted for multiple comparisons using Bonferroni correction. The diagnostic accuracy of GDF-15 for identifying frailty and sarcopenia dichotomously was assessed using receiver operating characteristic (ROC) analysis to determine the area under the curve (AUC), while Youden’s Index was applied to define the optimal predicted probability cut-off [38].

To further evaluate the validity of GDF-15 as a biomarker of frailty, logistic regression analyses were performed using the dichotomized GDF-15 variable (above vs. below the established cut-off). Odds ratios (ORs) with 95% confidence intervals (CIs) were calculated. Multivariable adjusted models (aORs) were constructed using covariates that differed significantly between frail and non-frail patients. Adjustments included age, history of falls, handgrip strength, gait speed, and SNAQ score.

Statistical significance level was set at p ≤ 0.05 (two-tailed). All statistical analyses were performed in SAS Studio.

## Results

This study included 429 patients referred to the outpatient clinic for fall assessment with complete records of CFS, sarcopenia, and GDF-15. Mean age was 79.6 years (SD 6.2); 64% were female, 15.6% had sarcopenia, and 24.9% were frail. Median GDF-15 plasma level was 1598 pg/mL for the total cohort, 1916 pg/mL for the group of sarcopenic patients, and 2252 pg/mL for the group of frail patients.

Table 1 summarizes the patient's demographic and anthropometric characteristics. No significant differences in age were observed between sarcopenic and non-sarcopenic patients, whereas frail patients were older than non-frail patients. The distribution of sexes was equal between groups. Frail and sarcopenic patients demonstrated significantly lower HGS, reduced STS performance, slower habitual GS, and higher SNAQ scores compared to their respective reference groups. Sarcopenic patients had lower BMI (p < 0.001), reduced muscle mass (p < 0.001), and were more likely to be frail (43.3% vs. 21.5%) (p < 0.001) compared to non-sarcopenic patients. Frail patients had experienced more falls in the past year (p < 0.001) and were more likely to be sarcopenic (p < 0.001) compared to non-frail patients (27.1% vs. 11.8%) (Table 1).

**Table 1:** Characteristics of mobility-limited older adults for the total cohort, and stratified according to frailty and sarcopenia status

	**Total cohort** **n = 429**	**Frail** **n = 107**	**p**	**Sarcopenia** **n = 67**	**p**	**Frail and sarcopenia** **n = 29**
Age (yr), mean (SD)	79.6 (6.2)	81.7 (6.4)	< 0.001	80.6 (6.2)	0.172	81.7 (6.1)
Female (%)	64.1	66.4	0.575	62.7	0.793	62.1
BMI (kg/m^2^), median [IQR]	25.3 [22.8–28.4]	25.4 [22.6–29.4]	0.947	21.9 [19.7–24.1]	< 0.001	21.2 [19.4–24.4]
SNAQ^a^ (%) Well-nourished Moderate malnutrition Severe malnutrition	83.5 6.1 11.3	69.0 8.7 22.3	<0.001	61.2 17.9 20.9	<0.001	41.4 24.1 34.5
Falls^b^ (%) No falls 1–3 fallls ≥ 4 falls	21.4 51.4 27.2	7.6 49.5 42.9	<0.001	20.9 41.8 37.3	9.19	6.9 48.3 44.8
Low HGS^c^ (%)	26.1	49.0	<0.001	57.6	<0.001	62.1
Low 30-s STS^d^ (%)	36.7	71.3	<0.001	59.3	<0.001	73.1
Low 5-reps STS^e^ (%)	29.9	61.4	< 0.001	55.8	<0.001	52.9
Low SMI^f^ (%)	26.3	28.0	0.646	100	<0.001	100
Low GS^g^ (%)	40.4	78.4	<0.001	55.7	0.008	70.4
Sarcopenia (%)	15.6	27.1	<0.001	N/A		N/A
Frailty (%)	24.9	N/A		43.3	<0.001	N/A
GDF-15 (pg/mL), median [IQR]	1598.2 [1007.4–2496.7]	2251.6 [1419.2–4112.9]	<0.001	1915.5 [1129.5–3515.2]	0.035	2160.7 [954.8–3458.8]

*Note:* BMI, body mass Index; GDF-15, growth differentiation factor 15; GS, gait speed; HGS, handgrip strength; SMI, skeletal muscle index; SNAQ, Short Nutritional Assessment Questionnaire; STS, sit-to-stand test

^a^n = 423 (frail n = 103, sarcopenia n = 67), ^b^n = 426 (frail n = 105, sarcopenia n = 67), ^c^n = 414 (frail n = 102, sarcopenia n = 66), ^d^n = 371 (frail n = 87, sarcopenia n = 59), ^e^n = 364 (frail n = 70, sarcopenia n = 52), ^f^n = 429 (frail n = 107, sarcopenia n = 67), ^g^n = 381 (frail n = 88, sarcopenia n = 61)

p < 0.05 indicate significant between-group differences

GDF-15 plasma levels were significantly higher in sarcopenic patients compared to non-sarcopenic patients (1916 pg/mL vs. 1569 pg/mL, p = 0.035). An identical trend was observed in frail patients compared to non-frail patients (2252 pg/mL vs. 1438 pg/mL, p < 0.001) (Table 1).

**Fig. 1:**
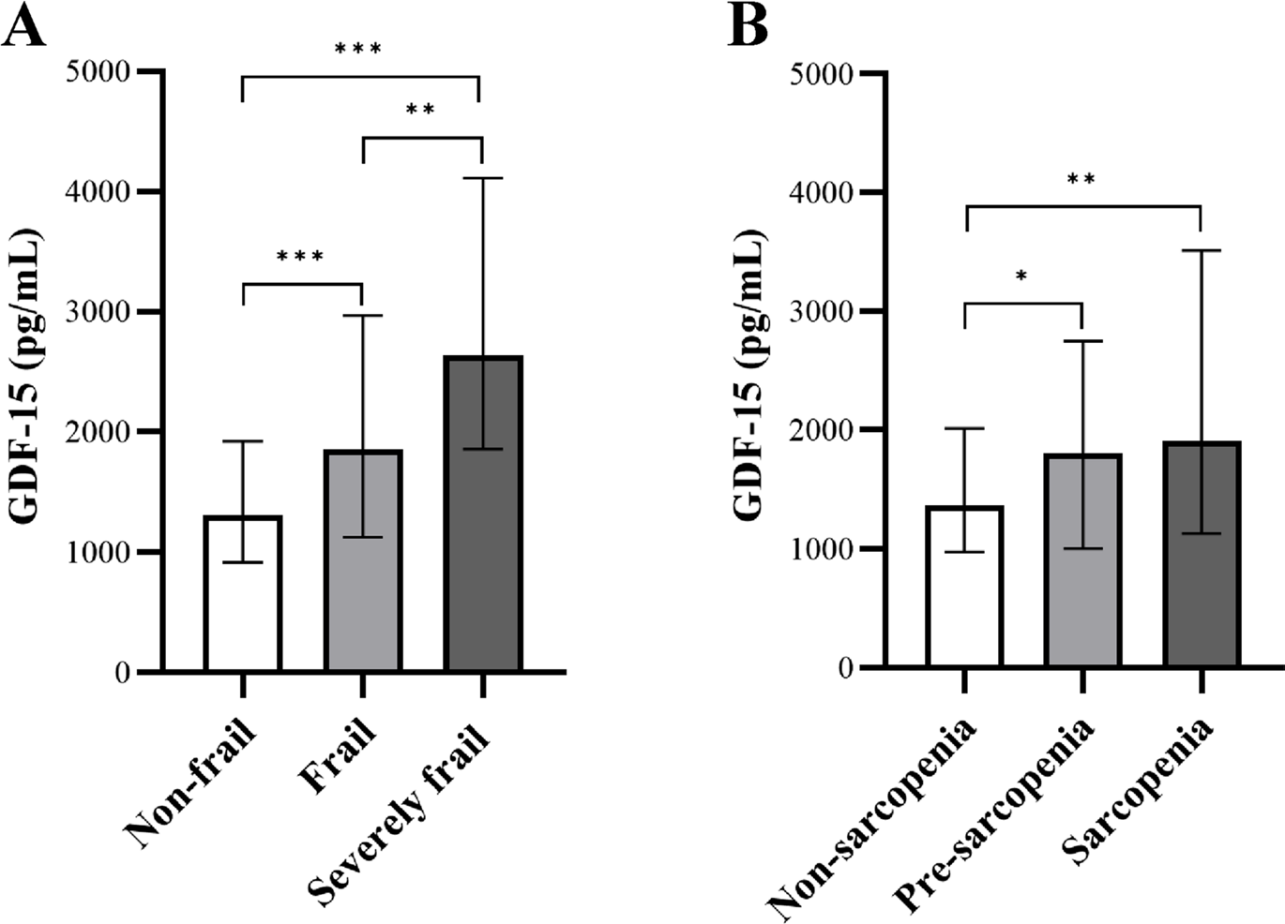
GDF-15 plasma levels across severity stages of **A**) frailty and **B**) sarcopenia (median (Q1–Q3)). Growth differentiation factor 15, GDF-15; * p ≤ 0.05, ** p < 0.01, *** p < 0.001

Figure 1 displays GDF-15 plasma levels obtained in non-frail, frail, and severely frail patients as well as in non-sarcopenia, pre-sarcopenia, and sarcopenia patients. The Kruskal–Wallis test revealed a significant difference in GDF-15 plasma levels between the three frailty groups, adjusted for multiple comparisons using the Bonferroni correction, with median levels of GDF-15 for the non-frail 1309 pg/mL, frail 1852 pg/mL, and severely frail 2644 pg/mL (p < 0.001) (Fig. 1 A).

No statistically significant differences in GDF-15 plasma levels were observed between pre-sarcopenic and sarcopenic patients. In contrast, GDF-15 levels differed significantly between non-sarcopenic and pre-sarcopenic patients (p = 0.017) and between non-sarcopenic and sarcopenic patients (p = 0.009): median GDF-15 plasma level was 1371 pg/mL in non-sarcopenic patients, 1806 pg/mL in pre-sarcopenic patients, and 1916 pg/mL in the sarcopenic patients (p = 0.004) (Fig. 1 B).

**Fig. 2:**
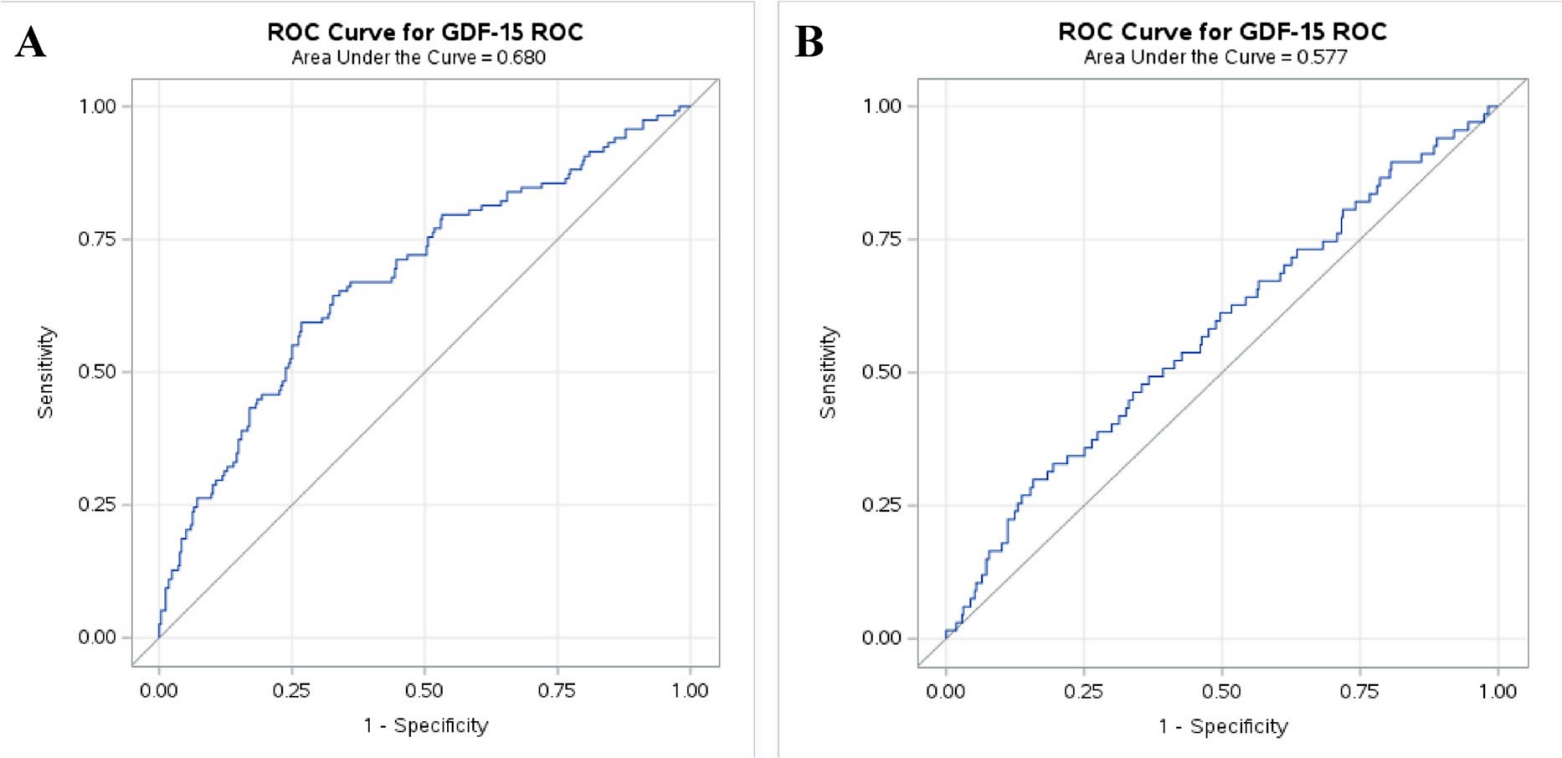
Receiver operator curve (ROC) analyses for diagnostic accuracy of GDF-15. (A) The area under the curve (AUC) for evaluating the potential for GDF-15 to identify frailty in mobility-limited older adults. AUC was 0.681 for frailty. (B) The area under the curve (AUC) for evaluating the potential for GDF-15 to identify sarcopenia in mobility-limited older adults. AUC was 0.577 for sarcopenia

The diagnostic accuracy of GDF-15 for frailty, as determined by AUC analysis, was 0.681 (95% CI: 0.623–0.739) for frailty, and 0.577 (95% CI: 0.501–0.653) for sarcopenia (Fig. 2). When stratified by sex, the AUC was 0.673 (95% CI: 0.597–0.750) for females and 0.706 (95% CI: 0.604–0.810) for males, indicating similar diagnostic accuracy in both groups (Supplementary Figure S1). The optimal frailty cut-off determined using Youden’s Index was 2047 pg/ml.

When dichotomized according to the outcome-related cut-off (2047 pg/mL), a total of n = 151 had GDF-15 levels above the threshold and a total of n = 278 had levels below. Logistic regression analyses showed that elevated GDF-15 was significantly associated with frailty. In the crude model, the odds ratio (OR) was 4.02 (95% CI: 2.54–6.36), and the association remained significant after full adjustment for age, history of falls, handgrip strength, gait speed, and SNAQ score, with an adjusted OR (aOR) of 2.91 (95% CI: 1.60–5.29) (Table 2).

**Table 2:** Multivariable logistic regression analyses of frailty by dichotomized GDF-15

	Frailty OR (95%CI), Crude model	Frailty OR (95%CI), Model a
GDF-15 Cut-off: 2046 pg/mL	4.02 (2.54–6.36)	2.91 (1.60–5.29)

CI, confidence interval; GDF-15, growth differentiating factor 15; GS, gait speed; HGS, handgrip strength; OR, odds ratio; SNAQ, Short Nutritional Assessment Questionnaire

High SNAQ was defined as a score ≥ 2. Low HGS defined as values < 27 kg and < 16 kg, for males and females, respectively. Low GS was defined as values ≤ 0.8 m/s (see text for details)

^a^Model adjusted for age history of falls, low HGS, low GS, and high SNAQ score

## Discussion

Frailty and sarcopenia are two interrelated yet distinct age-related conditions that impact the health and independence of older adults [3, 8]. Identifying sensitive and reliable biomarkers for these conditions may be considered critical for improving early detection, risk stratification, and improving their clinical applicability in managing and supporting mobility-limited older patients. To achieve this, the present study aimed to investigate the association between GDF-15 plasma levels and frailty and sarcopenia, respectively, in a cohort of mobility-limited older adults referred to an outpatient clinic for fall assessment.

The present data demonstrate a strong relationship between GDF-15 plasma levels and frailty severity, with higher GDF-15 levels observed in frail and severely frail individuals compared to their non-frail counterparts. Additionally, the diagnostic accuracy of GDF-15 for frailty was estimated by receiver operator curve analysis (AUC) to reach 0.681, i.e., to be of moderately high accuracy [39].

No significant difference in GDF-15 plasma level was observed between pre-sarcopenic patients and sarcopenic patients. In contrast, elevated levels of plasma GDF-15 were observed in pre-sarcopenia patients compared to non-sarcopenic patients, while also demonstrating substantially higher levels in sarcopenic patients compared to non-sarcopenic patients. These findings indicate a potential link between increasing GDF-15 plasma levels and the progression from non-sarcopenia to sarcopenia stages, indicating the potential role of plasma GDF-15 as a sensitive biomarker for detecting early physiological changes associated with pre-sarcopenia.

In the present study, GDF-15 plasma levels were found to be elevated in patients with frailty and appeared to be related to frailty severity in mobility-limited older adults, hence suggesting that GDF-15 represents a potential biomarker effective in identifying frailty in this population. These findings are aligned with increasing evidence suggesting that GDF-15 is intricately linked to age-related physiological decline and systemic vulnerability [40, 41]. Elevated GDF-15 levels are widely recognized to reflect increased systemic stress and inflammation [42, 43]. Frailty is characterized by a diminished capacity to respond to given physiological stressors, often driven by chronic low-grade inflammation [2, 3]. GDF-15 in the blood is found to be upregulated in response to myocellular stress, mitochondrial dysfunction, and increased proinflammatory activity [43, 44], suggesting GDF-15 serves as an integrative marker of these processes.

Specifically, GDF-15 has been linked to mitochondrial dysfunction, including impaired electron transport chain (ETC) activity and increased production of reactive oxygen species (ROS), which in turn promote cellular senescence and reduced tissue resilience in aging [13, 45]. Mitochondria are essential for cellular energy homeostasis, and dysfunction of the ETC can impair ATP synthesis, elevate oxidative stress, and activate pro-inflammatory and stress-related signaling pathways. In the context of chronic physiological stress, these changes may contribute to systemic decline and increased vulnerability, as observed in frailty.

Additionally, GDF-15 has been hypothesized to regulate overall energy balance, as it has been shown to reduce food intake and reduce appetite [46, 47], potentially explaining its association with weight loss – also a common feature of frailty. Chronic elevations of plasma GDF-15 have been shown to induce anorexia-like states [48], which could intensify the physical decline in frail older adults. These findings suggest that GDF-15 may not only reflect frailty-related processes, but may also actively contribute to their progression.

The diagnostic accuracy of GDF-15 plasma level for frailty, as indicated by the present observation of an AUC of 0.681, suggests a potential clinical role for GDF-15 in identifying frailty in mobility-limited older adults. Notably, the present optimal cut-off threshold for frailty (GDF-15 plasma level exceeding 2047 pg/mL) closely aligns with our previously reported threshold of 2166 pg/mL in acutely admitted older patients [24]. This alignment not only highlights the potential of GDF-15 as a reliable diagnostic biomarker of frailty but also suggests that its levels may remain relatively stable and less influenced by the acute physiological stresses associated with hospital admission. Such stability enhances its reliability and broad applicability as a more chronic biomarker across diverse settings and patient populations, from outpatient clinics to acute care environments.

Despite its biological plausibility, the extent to which GDF-15 directly drives frailty pathogenesis versus acting as a secondary marker of broader systemic dysfunction remains unclear. For instance, elevated GDF-15 plasma levels may arise as an adjunct by-product of chronic diseases commonly associated with frailty, such as cardiovascular diseases, chronic kidney disease, or cancer [18, 19, 22]. These conditions share overlapping inflammatory and stress-response pathways, which could confound the observed association between GDF-15 and frailty.

In the present study, we found that GDF-15 plasma levels were significantly higher in pre-sarcopenic and sarcopenic patients compared to non-sarcopenic patients, while there was no significant difference in GDF-15 plasma levels between pre-sarcopenic patients and sarcopenic patients (Fig. 1).

Previous studies have consistently reported statistically significant relationships between GDF-15 plasma levels and sarcopenia, supporting its potential role in the pathophysiology of age-related muscle decline [13, 24, 27]. In support of this notion, we have previously demonstrated that acutely admitted older patients with sarcopenia exhibit higher GDF-15 plasma levels compared to those without sarcopenia [24]. Moreover, systemic GDF-15 levels were found to increase with age in healthy males and females, while concurrently negatively associated with maximal muscle power [26], suggesting a potential role of GDF-15 in aging biology and mechanical muscle performance at old age. These findings align with other studies in healthy adults, which have linked higher circulating levels of plasma GDF-15 to lower muscle strength, reduced physical performance, and gait speed [49, 50].

In contrast, to the best of our knowledge, only a single study has reported that GDF-15 may not reliably predict sarcopenia risk. Specifically, Nga et al. (2021) found no significant difference in serum GDF-15 levels between older adults with and without sarcopenia [51]. However, their study was conducted in a community-dwelling Asian population using AWGS criteria, which may differ from the present cohort of mobility-limited patients referred for fall assessment. Additionally, differences in biomarker measurement and diagnostic criteria could contribute to the discrepancies in findings.

The present lack of any association between GDF-15 plasma levels and the transition from pre-sarcopenia to sarcopenia could be explained by the fact that these two groups differed only by the presence of low skeletal muscle mass, a parameter that may not *per se* be as strongly negatively affected by GDF-15 as muscle strength and functional capacity. Thus, the present data may reflect the established role of GDF-15 in systematic inflammation and stress responses, processes that are more closely associated with the decline in muscle strength than related to the reduction in muscle mass. Nevertheless, it remains unclear whether elevated GDF-15 levels *per se* reflect frailty and sarcopenia, or rather reflect underlying factors such as skeletal muscle loss, inflammation and/or elevated disease burden.

As discussed above, previous reports have linked elevated GDF-15 plasma levels to reduced muscle strength [49, 50], supporting the hypothesis that GDF-15 reflects early physiological changes associated with sarcopenia [24, 26]. Maximal muscle strength seems to be more dynamic and sensitive to systemic inflammation and oxidative stress, both of which are central to GDF-15’s biological functions. Conversely, muscle mass seems to decline more gradually and may involve more influential regulatory pathways, such as hormonal or metabolic factors, which may be less directly influenced by GDF-15.

The diagnostic accuracy of GDF-15 for predicting sarcopenia, as indicated by the present AUC of 0.577, suggests that GDF-15 only has low-to-moderate discriminatory ability in identifying sarcopenia within the present cohort. An AUC value near 0.50 indicates a performance comparable to random chances, which underlines the challenge of using GDF-15 as a stand-alone biomarker for sarcopenia diagnosis in mobility-limited older adults.

This finding contrasts with some previous reports showing associations between GDF-15 plasma levels and muscle strength or sarcopenia, raising questions about the potential factors contributing to these discrepancies [24, 26]. A possible explanation is that GDF-15, while reflecting certain physiological processes such as systemic inflammation or oxidative stress, may not sufficiently capture the complex multifactorial nature of sarcopenia, which involves both muscle strength and muscle mass [8]. In the present study, sarcopenia was defined based on both low muscle strength and low muscle mass as proposed by EWGSOP2, which may explain the limited discriminatory power of GDF-15, as its association with muscle strength is more well-established than its relationship with muscle mass.

Moreover, it is possible that the biological pathways influenced by GDF-15, such as those related to inflammation and myocellular stress, may not align closely with the specific physiological processes that lead to the loss of skeletal muscle mass in sarcopenia. Thus, while GDF-15 could serve as a marker for certain aspects of sarcopenia, it may not be sufficiently sensitive to effectively distinguish between individuals with and without sarcopenia.

### Strenghts and limitations

The present study contains several strengths. First, it included a relatively large cohort of mobility-limited older adults, which enhanced statistical power. Second, it was conducted in a well-defined cohort referred to an outpatient clinic for fall assessment, ensuring strong clinical relevance to a high-risk population. Third, the inclusion of both frailty and sarcopenia as diagnostic outcomes allowed for a comprehensive evaluation of GDF-15’s potential as a biomarker for two clinically significant age-related conditions.

A number of limitations may be mentioned as well. First, the cross-sectional design limits the ability to infer a causal relationship between GDF-15 plasma levels and the conditions of frailty and sarcopenia. Second, the sample size within the subgroups may not have been sufficient to allow differences between pre-sarcopenia and sarcopenia states to be detected. Third, the present analyses were not adjusted for potential confounding factors such as comorbidities (e.g., cardiovascular disease, chronic kidney disease, and cancer), which are known to elevate GDF-15 independently of frailty or sarcopenia. The lack of detailed information on these conditions represents a limitation of the present study, as it remains uncertain whether the increased GDF-15 levels observed in some patients *per se* would reflect frailty and sarcopenia specifically or emerge as a result of other disease processes. Fourth, the present study lacks direct measures of mitochondrial dysfunction or senescence-related pathways, which could have provided mechanistic insights into the observed associations. Including such data in future studies could help clarify the biological keystones of GDF-15’s role in frailty and sarcopenia. Finally, the relatively moderate AUC values observed in the present study indicate that while GDF-15 plasma level may serve as a useful biomarker of frailty, it is unlikely to function as a stand-alone diagnostic tool. Combining GDF-15 with additional biomarkers or clinical assessments may enhance its diagnostic accuracy.

## Conclusion

The present study demonstrated that GDF-15 plasma levels in a cohort of mobility-limited older adults were higher in pre-sarcopenia patients and sarcopenic patients compared to non-sarcopenic patients; however, while GDF-15 shows promise in understanding some of the underlying mechanisms of sarcopenia, its diagnostic accuracy as a standalone biomarker remains limited. Additionally, the present study demonstrated that GDF-15 plasma levels were strongly related to frailty severity and thus may be used as a potential biomarker to identify frailty in this population. Moreover, an optimal cut-off threshold for GDF-15 related to the presence of frailty was identified, and while the AUC analysis indicated a moderately strong diagnostic accuracy, its feasibility and ease of measurement in clinical settings would make it a promising candidate for further investigation as a diagnostic or prognostic tool. Nevertheless, additional research is necessary to validate these findings in diverse populations and across varying clinical contexts to establish its definitive role in frailty assessment.

## Supplementary Information

Below is the link to the electronic supplementary material.

ESM 1 (DOCX 91.8 KB)

## Data Availability

The data utilized in this study is available from the corresponding author [PH] upon reasonable request.
