# Supplementary material for: GDF-15 plasma levels are elevated in mobility-limited older adults with frailty and sarcopenia—results from the BIOFRAIL study
Source: GeroScience. 2025 Nov 25;48(2):1955–66. doi: 10.1007/s11357-025-01946-6 (PMC12972285; doi:10.1007/s11357-025-01946-6)
Supplement: Supplementary file 1 — (DOCX 91.8 KB) [file 11357_2025_1946_MOESM1_ESM.docx]

**Supplementary**

**Figure S1.** Receiver operator curve (ROC) analyses for diagnostic accuracy of GDF-15 in identifying frailty. (A) The area under the curve (AUC) for females (n=275), AUC = 0.673. (B) The area under the curve (AUC) for males (n=154), AUC = 0.706.
